# Cognitive Reserve and Well-Being Among Centenarians

**DOI:** 10.1093/geroni/igaa057.2821

**Published:** 2020-12-16

**Authors:** Peter Martin, Gina Lee, Yasuyuki Gondo, Leonard Poon

**Affiliations:** 1 Iowa State University, Ames, Iowa, United States; 2 Osaka University, Suita, Osaka, Japan; 3 University of Georgia, Athens, Georgia, United States

## Abstract

The purpose of this study was to assess cognitive reserve among centenarians and to evaluate whether reserve variables explain significantly more variance in cognitive functioning than education alone. Centenarians from the Georgia Centenarians Study were included. Results indicate that education, activity, social engagement, and engaged lifestyle significantly related to cognitive functioning. After adding cognitive reserve variables, the effect of education on cognitive functioning diminished. The overall model fit well, Χ2 (df=6) = 12.35, p = .06; CFI = .97, RMSEA = .067. Education indirectly related to cognitive functioning through occupational responsibility and engaged lifestyle. Social engagement directly related to cognitive functioning but also indirectly through activity levels. The four direct predictors (i.e., education, social engagement, activity, and engaged lifestyle) explained 33 percent of the variance in cognitive functioning. The results provide important information for cognitive reserve theories with implications for physical health and interventions that build cognitive reserves.

